# Influence of Radiotherapy Fractionation Schedule on the Tumor Vascular Microenvironment in Prostate and Lung Cancer Models

**DOI:** 10.3390/cancers12010121

**Published:** 2020-01-02

**Authors:** Karen Clément-Colmou, Vincent Potiron, Manon Pietri, Maëva Guillonneau, Emmanuel Jouglar, Sophie Chiavassa, Grégory Delpon, François Paris, Stéphane Supiot

**Affiliations:** 1 Centre de Recherche en Cancérologie Immunologie Nantes Angers (CRCINA), Institut National de Santé et de la Recherche Médicale (INSERM) UMR U1232, Centre National de la Recherche Scientifique (CNRS) ERL 6001, Université de Nantes, 44007 Nantes, France; karen.clement-colmou@ico.unicancer.fr (K.C.-C.); vincent.potiron@univ-nantes.fr (V.P.); manon.pietri@univ-nantes.fr (M.P.); Maeva.Guillonneau@univ-nantes.fr (M.G.); emmanuel.jouglar@ico.unicancer.fr (E.J.); sophie.chiavassa@ico.unicancer.fr (S.C.); Gregory.Delpon@ico.unicancer.fr (G.D.); Francois.Paris@univ-nantes.fr (F.P.); 2 Laboratoire de Biologie des Cancers et de Théranostic (LabCT), Institut de Cancérologie de l’Ouest, 44800 Saint-Herblain, France; 3 Service de Radiothérapie, Institut de Cancérologie de l’Ouest, 44800 Saint-Herblain, France; 4 Service de Physique Médicale, Institut de Cancérologie de l’Ouest, 44800 Saint-Herblain, France

**Keywords:** radiotherapy, fractionation, microenvironment, vasculature, hypoxia, stereotactic radiotherapy

## Abstract

Background. The tumor vasculature acts as an interface for the primary tumor. It regulates oxygenation, nutrient delivery, and treatment efficacy including radiotherapy. The response of the tumor vasculature to different radiation doses has been disparately reported. Whereas high single doses can induce endothelial cell death, improved vascular functionality has also been described in a various dose range, and few attempts have been made to reconcile these findings. Therefore, we aimed at comparing the effects of different radiation fractionation regimens on the tumor vascular microenvironment. Methods: Lewis lung and prostate PC3 carcinoma-derived tumors were irradiated with regimens of 10 × 2 Gy, 6 × 4 Gy, 3 × 8 Gy or 2 × 12 Gy fractions. The tumor vasculature phenotype and function was evaluated by immunohistochemistry for endothelial cells (CD31), pericytes (desmin, α-SMA), hypoxia (pimonidazole) and perfusion (Hoechst 33342). Results: Radiotherapy increased vascular coverage similarly in all fractionation regimens in both models. Vessel density appeared unaffected. In PC3 tumors, hypoxia was decreased and perfusion was enhanced in proportion with the dose per fraction. In LLC tumors, no functional changes were observed at *t* = 15 days, but increased perfusion was noticed earlier (*t* = 9–11 days). Conclusion: The vascular microenvironment response of prostate and lung cancers to radiotherapy consists of both tumor/dose-independent vascular maturation and tumor-dependent functional parameters.

## 1. Introduction

Independently of the intrinsic nature of cancer cells, the tumor microenvironment influences tumor growth, progression, and response to treatments [1]. In this regard, special emphasis has been placed on the vascular microenvironment [2]. As the result of tumor excessive signals, the tumor vasculature is often disorganized, poorly mature, and less efficient [3]. Strategies to restore closer-to-normal function have been proposed [4]. In certain conditions, anti-angiogenic drugs normalize the vasculature by improving pericyte coverage, basal lamina continuity and vascular permeability [5]. 

We and others have shown that fractionated radiotherapy (RT) induces normalization of the tumor vasculature, characterized by enhanced pericyte coverage, blood perfusion, and reduction of intra-tumoral hypoxia [6,7,8,9]. In a time-course experiment using 10 × 2 Gy, we originally found that increased pericyte coverage starts at one week and is more visible at two weeks [7]. Similarly, Lan et al. [10] have used 3 irradiations of 12 Gy separated by one week (3 × 12 Gy) and also found increased vascular coverage starting at 1 week and more pronounced at 2 weeks. Chen et al. [6] have reported similar findings at 3 weeks but have not explored earlier time points. Lastly, in the non-tumoral brain, Burrell et al. [11] reported the recruitment of pericytes (among many BMDCs) from 1 week to 1 month after RT. Collectively, these data indicate that vascular normalization takes place from 1–3 weeks after the initiation of treatment [7,10,12]. These findings appear of clinical interest considering the beneficial role of oxygen in the efficacy of radiotherapy. However, the vascular consequences of high dose-per-fraction irradiation are not so clearly understood. Whereas vascular maturation has been reported after fractions up to 3 × 12 Gy [10], other studies have found increased hypoxia, reduction in vessel density and even endothelial cell death [13,14,15,16,17]. Collectively, it is difficult to interpret whether the response to radiotherapy of the tumor vasculature depends on a dose threshold or whether it relies on specific tumor model parameters. 

Here, we aimed at comparing the effects of increasing dose-per-fraction RT on the phenotype and functionality of the vascular microenvironment. Different RT schedules that are clinically conceivable were designed. Moreover, dose-per-fraction spanned around the theoretical 7 Gy threshold that has been proposed earlier to initiate endothelial cell death [13,16]. Two independent tumor models were used, the Lewis lung and prostate PC3 carcinomas. Established subcutaneous tumors were exposed to localized X-ray irradiation for two consecutive weeks with different planning: 10 × 2 Gy, 6 × 4 Gy, 3 × 8 Gy and 2 × 12 Gy. Microvascular density (CD31), pericyte coverage (desmin, α-SMA), hypoxic zones (pimonidazole), and perfusion (Hoechst 33342) were determined by immunohistochemistry and compared between the different methods. 

## 2. Results

### 2.1. Enhancement of Tumor Control by High Dose-Per-Fraction Is Tumor-Dependent

**Figure 1:**
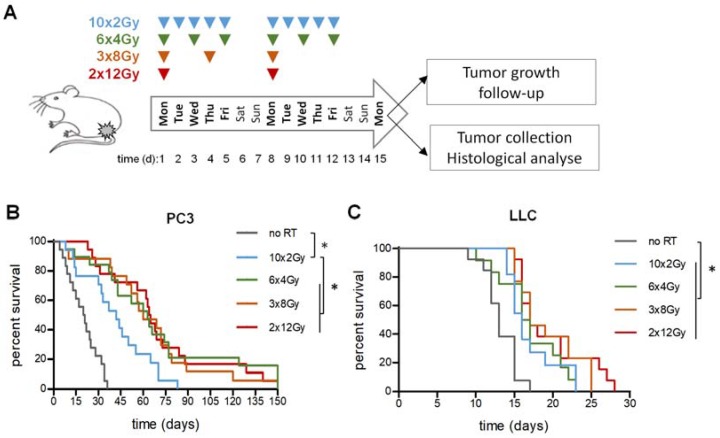
Tumor control in response to radiotherapy (RT) fractionation schedule. (**A**) Experimental calendar of fractionation. (**B**,**C**) Kaplan–Meier survival estimates of PC3 (**B**) and LLC (**C**) tumor-bearing animals. Established tumors were irradiated as indicated and animals were counted dead when the tumor volume reached 2000 mm^3^. Experiments were done twice with a total of *n* ≥ 15 per group. * indicates *p* < 0.05.

First, to compare the anti-tumor effects of different irradiation schedules, established tumors were locally irradiated with either 10 × 2 Gy fractions, 6 × 4 Gy fractions, 3 × 8 Gy fractions, or 2 × 12 Gy fractions (Figure 1A). The time to achieve a 2000 mm^3^ volume was recorded for each individual and plotted as survival Kaplan–Meier curves. As expected, RT significantly prolonged survival in all fractionation schedules compared to non-irradiated controls (Figure 1B,C; Supplementary Figure S1). For PC3 tumors, the medians were 20 d for controls, 42 d for 10 × 2 Gy, 63 d for 6 × 4 Gy, 60 d for 3 × 8 Gy and 64 d for 2 × 12 Gy. Compared to conventional 10 × 2 Gy, 6 × 4 Gy, 3 × 8 Gy, and 2 × 12 Gy were all more efficient, although not differently from one another (Figure 1B). For LLC tumors, medians were 13 d for controls, 16 d for 10 × 2 Gy, 16.5 d for 6 × 4 Gy, 17 d for 3 × 8 Gy, and 17 d for 2 × 12 Gy (Figure 1C). No RT schedule was significantly more efficient than the others (Figure 1C). We do not, however, rule out that minor differences could be demonstrated using a substantially higher number of animals.

Thus, fractionation had less differential impact on the fast-proliferating, LLC model than on the PC3 model.

### 2.2. RT Induces Vascular Coverage Independently of the Fractionation Schedule

**Figure 2:**
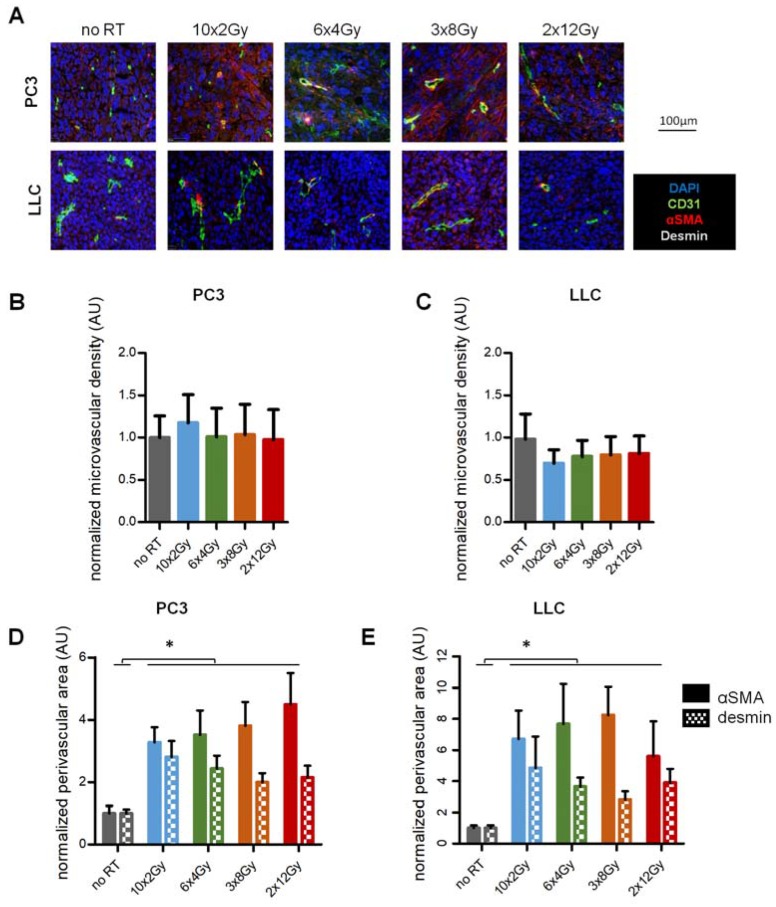
Tumor vascular phenotype in response to RT fractionation schedule. (**A**) Immunohistological staining for pericyte coverage (αSMA, desmin) around tumor vessels (CD31) two weeks after RT initiation in PC3 and LLC tumors. (**B**,**C**) Quantification of vessel density in PC3 (**B**) and LLC (**C**) tumors at day 15. (**D**,**E**) Quantification of vascular mural coverage (αSMA: plain, desmin: squared) in PC3 (**D**) and LLC (**E**) tumors at day 15. Images and analysis represent two independent experiments with a total ≥18 tumors per point. * indicates *p* < 0.05.

Next, we assessed how RT fractionation affects the tumor vasculature. First, microvessel density (MVD) was assessed two weeks (d 15) from the first irradiation. No significant changes were noted in PC3 and LLC tumors, neither compared to non-irradiated tumors nor between the different RT schedules (Figure 2A–C). MVD were 100 (no RT), 117 (10 × 2 Gy), 101 (6 × 4 Gy), 103 (3 × 8 Gy), and 97 (2 × 12 Gy) microvessels/mm^2^ in PC3 tumors, and 180 (no RT), 120 (10 × 2 Gy), 138 (6 × 4 Gy), 136 (3 × 8 Gy), and 144 (2 × 12 Gy) microvessels/mm^2^ in LLC tumors. 

Non-irradiated tumor vessels were poorly covered by pericytes. In contrast, all RT regimens increased coverage of the vessels by α-SMA and desmin-positive pericytes (Figure 2A,D,E). In PC3, α-SMA was upregulated between 3.3 (10 × 2 Gy) and 4.5 fold (2 × 12 Gy) and desmin between 2.0 (3 × 8 Gy) and 2.81 (10 × 2 Gy). In LLC, α-SMA was upregulated between 5.6 (2 × 12 Gy) and 8.3 (3 × 8 Gy) fold and desmin between 2.8 (3 × 8 Gy) and 4.8 (10 × 2 Gy). However, no statistical difference was noted between the RT fractionation schedules. Thus, RT consistently led to vascular coverage two weeks after the first irradiation, but regardless of the fractionation schedule.

### 2.3. Enhancement of Perfusion and Reduction of Hypoxia Correlate with Dose-Per-Fraction but Are Tumor-Dependent

**Figure 3:**
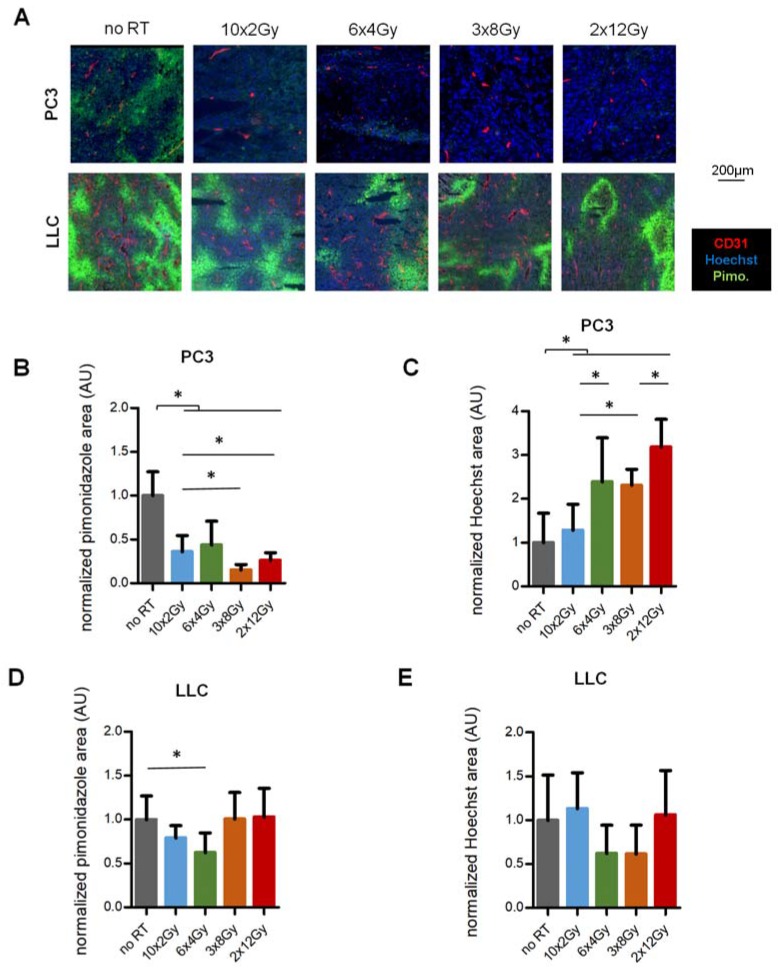
Tumor hypoxia and perfusion in response to RT fractionation schedule. (**A**) Immunohistological staining for hypoxia (pimonidazole) and perfusion (Hoechst 33342) two weeks after RT initiation in PC3 and LLC tumors. (**B**,**C**) Quantification of hypoxia in PC3 (**B**) and LLC (**C**) tumors at day 15. (**D**,**E**) Quantification of Hoechst perfusion in PC3 (**D**) and LLC (**E**) tumors at day 15. Images and analysis represent two independent experiments with a total ≥18 PC3 and ≥15 LLC tumors per point. * indicates *p* < 0.05.

To pursue these findings, we assessed whether vascular maturation translates into an improvement of functional parameters: increase of tumor perfusion or reduction of hypoxia. Hypoxia was determined by intra-tumoral pimonidazole adducts and perfusion was evaluated by distribution of Hoechst 33342 injected intravenously [9]. In PC3 tumors, the average baseline hypoxic surface was 9% of total tissue area (Figure 3A). Hypoxic surface was reduced with all RT schedules (10 × 2 Gy: −64%, 6 × 4 Gy: −57%, 3 × 8 Gy: −74% and 2 × 12 Gy: −85%; *p* < 0.0001) (Figure 3A,B). Moreover, stronger reduction was observed with the high dose-per-fraction protocols (10 × 2 Gy vs 3 × 8 Gy: *p* = 0.02). Conversely, perfusion was increased in all RT schedules vs non-irradiated tumors (10 × 2 Gy: +28%, 6 × 4 Gy: +139%, 3 × 8 Gy: +130% and 2 × 12 Gy: +218%; *p* < 0.0001) (Figure 3A,C). Also, perfusion was grossly increased as a function of the dose-per-fraction (10 × 2 Gy vs. 6 × 4 Gy, *p* = 0.05, 10 × 2 Gy vs 3 × 8 Gy: *p* = 0.003; 3 × 8 Gy vs. 2 × 12 Gy: *p* = 0.0003). At baseline, LLC tumors were more hypoxic than PC3 tumors, with an average pimonidazole-positive area of 29% in the non-irradiated controls (Figure 3A,D). Hypoxia reduction in the LLC tumors was heterogeneous, with a 21% reduction in 10 × 2 Gy (NS) and a 38% reduction in 6 × 4 Gy compared to control (*p* = 0.03), but no overall significance between all RT schedules and controls. In addition, there was no consistent change in Hoechst 33342 perfusion compared to control or between RT schedules in our conditions (Figure 3A,E). Interestingly, when comparing three tumor models with the same schedule (10 × 2 Gy), we observed that hypoxia reduction after RT negatively correlates with tumor proliferation (Supplementary Figure S2). Moreover, tumor perfusion inversely correlated with tumor cell density (Supplementary Figure S3A,C) but not with desmoplasia (Supplementary Figure S3B,C). Together, these results indicate that RT regulates perfusion/hypoxia with effects of the dose-per-fraction, but this is tumor-dependent (Figure 3C).

### 2.4. Enhancement of Vascular Function Occurs Transiently in Weakly-Responsive LLC Tumors

**Figure 4:**
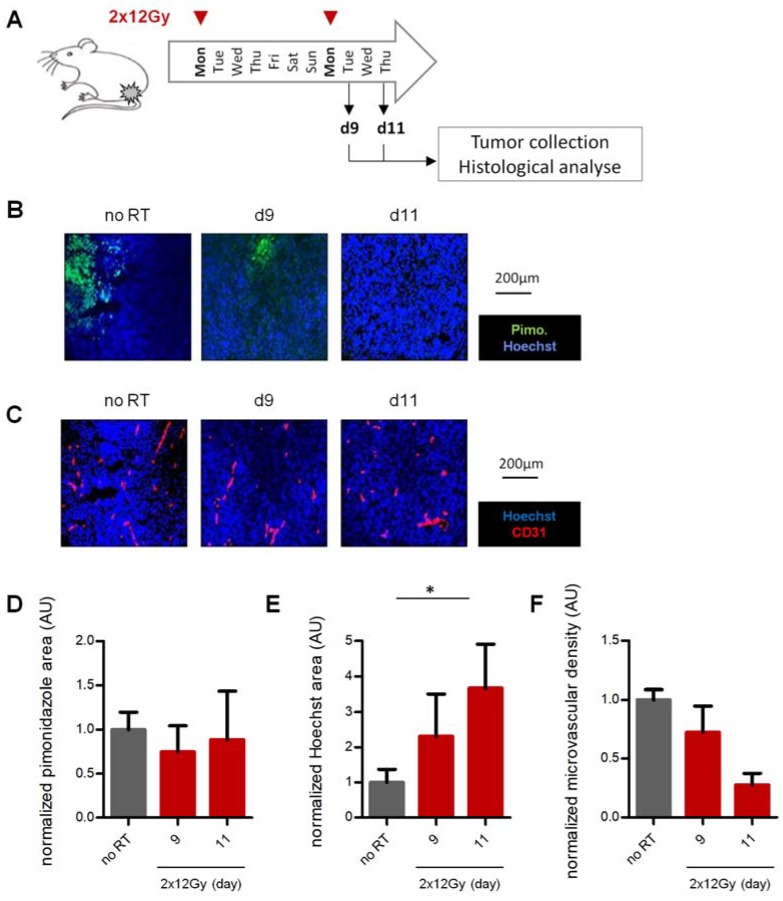
Time window of vascular changes after 2 × 12 Gy in LLC tumors. (**A**) Fractionation and collection schedule. (**B**) Immunohistological staining for hypoxia (pimonidazole, **B**), vessels and perfusion (CD31 and Hoechst 33342, **C**) in function of time after RT initiation in LLC tumors. (**D**–**F**) Quantification of hypoxia (pimonidazole, **D**), perfusion (Hoechst 33342, **E**) and vessel density (**F**) after RT initiation in LLC tumors. Controls were collected at d9. Images and analysis represent ≥7 tumors per point. * indicates *p* < 0.05.

Vascular normalization has been originally described as a temporary window after VEGFR2 blockade [18]. Because LLC tumors are more rapidly growing than PC3, we considered the possibility that rapid repopulation could minimize the functional vascular response. LLC tumors were irradiated with 2 × 12 Gy as previously, but tumors were collected at earlier timepoints after the last irradiation (Figure 4A). Hypoxia was non-significantly reduced at day 9–11 (Figure 4B,D). More importantly, perfusion was statistically increased at day 11, (*p* = 0.04, Figure 4C,E), unlike day 15 observations (Figure 3E). Interestingly, MVD was inversely regulated with a decrease at day 11 (*p* = 0.006, Figure 4C,F).

Thus, LLC tumors exhibited a vascular functional response to high dose-per-fraction 2 × 12 Gy schedule, but this was visible only short-term after the last RT.

## 3. Discussion

Vascular normalization is an alternative strategy to vascular destruction with the benefit of improving intra-tumoral oxygenation [4]. This is of interest for radiotherapy whose efficacy originates in part from oxygen-dependent biochemical reactions. Radiotherapy induces vascular normalization in certain conditions, but reports in the literature have yet to draw any consensus because of their inherent methodological disparities. This work aimed at comparing the effects of RT fractionation schedules on the tumor vascular microenvironment. We found that pericyte coverage is a consistent response to RT that occurs independently of the tumor model and of the dose-per-fraction. In contrast, hypoxia reduction and increased perfusion were dose-per-fraction and tumor-dependent. 

Increased pericyte coverage has been shown after fractionated RT, with some evidence for contribution of bone-marrow-derived cells [6,7,8,9,11]. However, observations after high dose RT were more heterogeneous [10,13,14,19]. Whether there is a relationship between dose-per-fraction and perivascular coverage had not been investigated in comparable settings [17]. In both our models, pericyte coverage was increased independently of the fractionation regimen. In addition, pericyte recruitment was observed similarly in conditions with different tumor control and alpha/beta ratios. Thus, pericyte recruitment is probably a host response that is independent from the tumor characteristics and radiosensitivity.

The role and regulation of pericytes in cancer biology draws increasing attention. At the physiological state, pericytes are involved in blood flow, histo-hematological barrier and tissue homeostasis [20]. In preclinical tumor models, disrupting pericyte coverage has been associated with enhanced hypoxia and metastasis [21,22], and conversely, increasing vessel coverage led to improved perfusion and drug delivery, lower metastasis incidence and infiltration with anti-tumor immune cells [23]. In this regard, we have shown that RT-induced vascular remodeling increases drug distribution [9]. Recently, the pericyte phenotype was linked to specific chemotherapy response in cancer patients [24].

More generally, interfering with vessel integrity is associated with hypoxia and metastasis [25,26]. This is of particular concern regarding radiotherapy since 7 Gy RT has been shown to trigger endothelial cell apoptosis [13]. Whether high RT fractions lead to vessel death has been disparately reported. In our conditions, MVD was apparently stable even after 2 × 12 Gy. Nevertheless, transitory reduction was observed, suggesting that endothelial cell death may occur at this dose but is rapidly compensated by the formation of new vessels. Indeed, Chen et al. have seen decreased vessel density in a similar context. Also, endothelial cell death is not necessary detrimental to blood vessel function below a certain extent [27]. Contrary to normal tissues where regrowth is absent, we presume that endothelial destruction after RT may not systematically translate into long-lasting effects in tumors. It appears that vascular normalization after RT can happen even with concomittant vessel destruction. One hypothesis is that anti-tumor treatments preferentially prune poorly covered/functional vessels [28]. Thus, vascular destruction and vascular normalization are not exclusive phenomena.

The tissue oxygen levels are the result of a complex balance between oxygen consumption and supply. Hypoxia reduction is not consistently proportional to perfusion enhancement, which implies that tumor repopulation may modulate intra-tumoral hypoxia. In parallel, a decrease in tumor cell density or extracellular matrix composition may facilitate blood perfusion by relieving mechanical compression of blood vessels, and by reinstating a fluid pressure balance favorable to interstitial diffusion [29]. Moderately growing PC3 showed differential growth sensitivity to fractionation (as highlighted by EQD2s, Supplementary Figure S4), in contrast to LLC. These findings are in agreement with a weak dependency on fractionation for rapidly growing tumors with high alpha/beta ratio [30], such as LLC. Despite a constant increase of vascular coverage after RT, effects at the functional level were variable. In the PC3 model where high-dose-per fraction RT was more efficient than conventional 10 × 2 Gy, hypoxia and perfusion were dose-dependent. In the LLC model with rapid repopulation, the perfusion increase after 2 × 12 Gy was limited in time (3–7 days). Of note, other studies [10] and other experiments in our hands [9] have been able to observe this up to 7 days, probably because of variations in tumor growth rate. Yet, our data show that perfusion and hypoxia were affected, at least partially, in proportion with tumor control, consistent with previous reports [12]. This would explain that the alpha/beta ratio remains approximately valid in vivo despite the phenotypic response of the vascular microenvironment [31]. Moreover, this reinforces the theory that patients with lower repopulation after RT would exhibit greater tumor reoxygenation.

Overall, vascular normalization draws increasing attention and raises opportunities for patient treatment. Whereas little has been made to exploit this phenomenon in the field of radiotherapy, evidence has emerged that chemotherapy benefits most to cancer patients who display vascular normalization [32]. Because of the importance of oxygenation for radiotherapy efficacy, further work should determine how to best take advantage of radio-induced vascular remodeling.

## 4. Materials and Methods

### 4.1. Cell Lines and Culture

Prostate adenocarcinoma PC3 cells (PC3-luc, Caliper Life Sciences, Villepinte, France) were cultivated in RPMI 1640 medium with 10% FBS in standard 37 °C/5% CO_2_ conditions. Lewis lung carcinoma cells (LL/2, LGC Standards, Molsheim, France) were cultivated in DMEM medium in standard conditions.

### 4.2. Animal Experiments

Tumors were generated by subcutaneous injections in the back left leg for RT ballistic purposes. Xenogeneic PC3 tumors were induced in 6–8 weeks old male NMRInu mice (Janvier, Saint Berthevin, France) with 2 × 10^6^ cells in 50 µL of medium without FBS 21 days before RT. Syngeneic LLC tumors were induced in 6–8 weeks old female C57BL/6 mice (Janvier) with 10^6^ cells 10 days before RT. Each group was composed of 7–10 animals per fractionation schedule, and each experiment was performed twice. Tumors were measured with a caliper and volume was estimated with the V = 0.5 × a × b^2^ formula [33]. For immunohistochemistry, animals were sacrificed and tumors were excised 14 days after the first dose of RT. For tumor growth, animals were euthanized when the tumor reached 2000 mm^3^. All experiments were carried out in accordance with the European Council Directive 2010/63/UE and approved by the local Animal Care and Use Committee (C2EA-06).

### 4.3. Radiotherapy

Animal irradiations were performed using a CP-160 X-ray irradiator (Faxitron, Lincolnshire, IL, USA) with an accelerating voltage of 160 kV and a dose rate of 1.3 Gy/min. Mice were immobilized in custom-made contention tubes. RT was centered on the tumor and the rest of the body was covered by lead shields to minimize irradiation to normal tissues. 

### 4.4. In Vivo Hypoxia and Perfusion

Hypoxia and perfusion were determined using extrinsic markers that were administered in saline buffer. For hypoxia, 100 µL of 70 mg/mL pimonidazole (Hypoxyprobe, Burlington, MA, USA) were injected intraperitoneally 90 min before sacrifice. For perfusion, 100 µL of 5 mg/ml Hoechst 33342 (Life Technologies, Saint Aubin, France) were injected intravenously 4 min before tissue collection.

### 4.5. Immunohistochemistry

Tumors were excised immediately after sacrifice and flash-frozen in OCT medium (Sakura Finetech SAS, Villeneuve d’Ascq, France). Immunohistochemistry was performed using previously reported procedures [7]. The following primary antibodies were used: Rat anti-mouse CD31 (BD Biosciences, Le Pont de Claix, France), Cy3-conjugated mouse anti-αSMA (Sigma-Aldrich, Saint-Quentin Fallavier, France), rabbit anti-mouse desmin (Life Technologies), FITC-conjugated anti-pimonidazole (Hypoxyprobe). The secondary antibodies were: Alexa^647^-conjugated goat anti-rabbit and Alexa^488^-conjugated goat anti-rat (Life Technologies). Slides were mounted in Prolong Gold with DAPI (Life Technologies) except for the perfusion assay. Images were acquired using a Nanozoomer HT slide scanner (Hamamatsu Photonics, Massy, France) at 20× or 40× resolution.

### 4.6. Image Analysis

Each image was recorded under native conditions. Analysis were performed on tiff files using ImageJ and custom-made macros, as previously described [7]. In brief, segmentation was done based on a negative control and neighboring background. Vessels were counted as distinct CD31+ objects. A pericyte coverage index was obtained by measuring the α-SMA and desmin positive area in a 2 µm perimeter around the CD31-positive area. Hypoxic and perfused areas were pimonidazole and Hoechst 33342 positive zones, respectively, over total tissue area.

### 4.7. Statistics

Survival analyses were done by the Kaplan–Meier method. Group comparisons were performed by ANOVA, or with Kruskal–Wallis test when Barlett’s test for standard deviation was significant. Statistical calculations were performed with Prism software (version 5.02, GraphPad, La Jolla, CA, USA). Differences were considered significant when *p* ≤ 0.05.

## 5. Conclusions

In conclusion, this study demonstrates that RT induces vascular remodeling independently of the tumor model and of the dose-per-fraction. However, the phenotypic vascular changes after RT do not necessarily translate into functional effects (hypoxia, perfusion). Instead, the perfusion/hypoxia responses depend on intrinsic tumor parameters such as radiosensitivity or proliferation rate. Because of this, the alpha/beta appears as the main correlate of tumor behavior in response to RT.

## Supplementary Materials

The following are available online at https://www.mdpi.com/2072-6694/12/1/121/s1, Figure S1: Tumor growth of PC3 and LLC after RT. Established tumors were irradiated as indicated and tumor volume was followed. Figure S2: Comparison of tumor growth rate (doubling time) and hypoxia reduction following RT. Figure S3: Cellular density and fibrosis in PC3 and LLC tumor cells. Figure S4: Radiosensitivity of PC3 and LLC tumor cells.
