# Supplementary material for: Influence of Radiotherapy Fractionation Schedule on the Tumor Vascular Microenvironment in Prostate and Lung Cancer Models
Source: Cancers (Basel). 2020 Jan 2;12(1):121. doi: 10.3390/cancers12010121 (PMC7017121; doi:10.3390/cancers12010121)
Supplement: Supplementary file 1 [file cancers-12-00121-s001.pdf]

cancers

MDPI

Article

Influence of Radiotherapy Fractionation Schedule on the Tumor Vascular Microenvironment in Prostate and Lung Cancer Models

Karen Clément-Colmou, Vincent Potiron, Manon Pietri, Maëva Guillonneau, Emmanuel Jouglar, Sophie Chiavassa, Grégory Delpon, François Paris and Stéphane Supiot

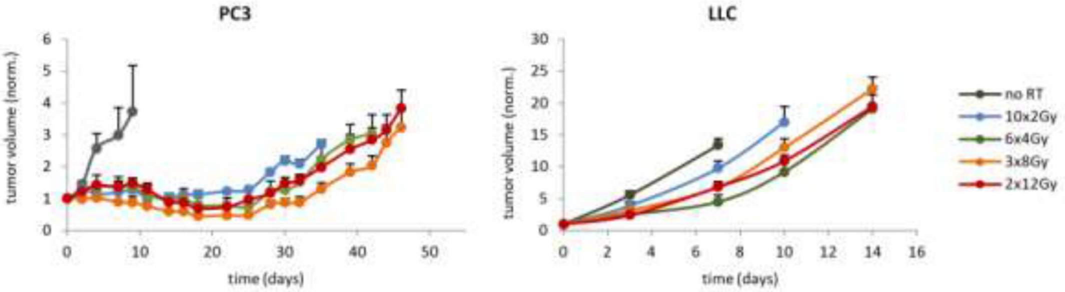

Figure S1. Tumor growth of PC3 and LLC after RT. Established tumors were irradiated as indicated and tumor volume was followed. Representative results of one of two independent experiments. Note that average calculation is no longer possible whenever one tumor in the group reaches the 2000 mm3 limit.

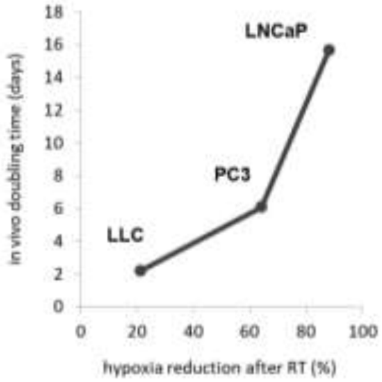

Figure S2. Comparison of tumor growth rate (doubling time) and hypoxia reduction following RT. Doubling times were estimated using exponential curve fits using the average of control tumors growth. Hypoxia reduction was measured two weeks after 10x2Gy. LNCaP data were calculatedfrom ref 9.

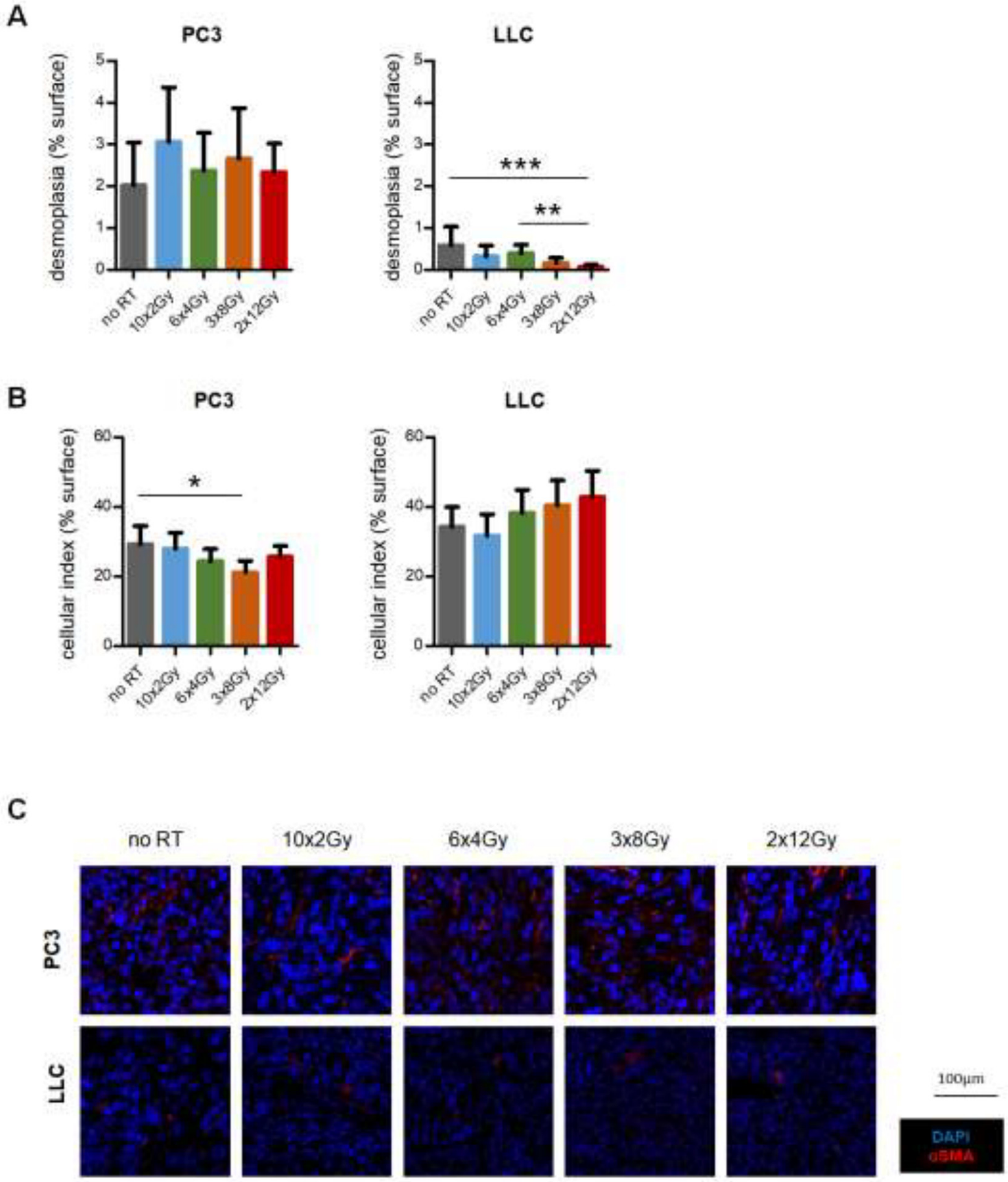

Figure S3. Cellular density and fibrosis in PC3 and LLC tumor cells. (**A**) Non perivascular SMA+ cells (desmoplasia) in tumors as a function of the RT schedule. (**B**) Cell nuclei (DAPI) density. (**C**) Representative images of A,B. Images and analysis represent two independent experiments with a total ≥15 tumors per point.

Desmoplasia, a mechanism of fibrosis, can occur in neoplasms at the basal state or as a treatment-induced reaction. Secretion of a dense and rigid extracellular matrix can in turn lead to vessel compression and restrict blood perfusion (ref 29 of the manuscript). We analyzed the extent of non perivascular smooth-muscle actin (SMA) positive cells, for identifying fibroblasts. We have not found that increased desmoplasia is able to explain the low response of the LLC tumors in term of perfusion. LLC tumors exhibited much less fibrosis than PC3, and this was further reduced upon irradiation (Fig. S3A, C). Instead, the cellular density after RT correlated with perfusion/hypoxia responses in both models (Supplementary Figure S3B,C).

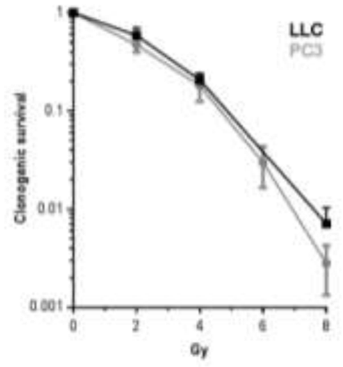

Figure S4. Radiosensitivity of PC3 and LLC tumor cells. Clonogenic curves of PC3 and LLC (tumor cells irradiated with increasing doses. The α and β parameters were calculated using GraphPad Prism by fitting curves using the formula: $S = e -(\alpha D+\beta D2)$, where S is the surviving fraction and D the dose of RT. Equivalent total dose in 2 Gy fraction (EQD2) for PC3 and LLC tumor cells, were calculated based on the α/β ratios.

For the PC3 model, the calculated α/β ratio was 2.1 Gy (Supplementary Figure S4), consistent with usual values for prostate carcinoma. The hypothetical equivalent doses in 2 Gy fractions (EQD2) were 20 Gy for 10 × 2Gy, 35,7 Gy for 6 × 4Gy, 59,1 Gy for 3 × 8Gy and 82,5 Gy for 2 × 12Gy. For the LLC model, The calculated α/β ratio was 8.2 Gy (Supplementary Figure S4), consistent with usual values for lung carcinoma. The EQD2 were 20Gy for 10 × 2Gy, 28,8 Gy for 6 × 4Gy, 38,3 Gy for 3 × 8Gy and 47,8 Gy for 2 × 12Gy.

© 2020 by the authors. Submitted for possible open access publication under the terms and conditions of the Creative Commons Attribution (CC BY) license (http://creativecommons.org/licenses/by/4.0/).
